# Plasmonic sensing using Babinet’s principle

**DOI:** 10.1515/nanoph-2023-0317

**Published:** 2023-09-27

**Authors:** Joseph Arnold Riley, Michal Horák, Vlastimil Křápek, Noel Healy, Victor Pacheco-Peña

**Affiliations:** School of Mathematics, Statistics and Physics, Newcastle University, Newcastle Upon Tyne, NE1 7RU, UK; School of Engineering, Newcastle University, Newcastle Upon Tyne, NE1 7RU, UK; Central European Institute of Technology, Brno University of Technology, Purkyňova 123, 612 00, Brno, Czech Republic; Institute of Physical Engineering, Brno University of Technology, Technická 2, 616 69, Brno, Czech Republic

**Keywords:** plasmonics, nanoparticles, plasmonic dimers, Babinet, dielectric sensing, nanoantennas

## Abstract

Developing methods to sense local variations in properties of nearby materials, such as their refractive index and thickness, are important in numerous fields including chemistry and biomedical applications. Localized surface plasmons (LSPs) excited in plasmonic nanostructures have been demonstrated to be useful in this context due to the spectral location of their associated resonances being sensitive to changes in the environment near the plasmonic structures. This manuscript explores Babinet’s principle by exploiting LSP resonances excited in complementary metal-dielectric cylindrical plasmonic structures (plasmonic particle-dimers and aperture-dimers in our case). Both plasmonic structures are evaluated numerically and experimentally using electron energy loss spectroscopy (EELS), providing a full physical understanding of the complementary nature of the excited LSP resonances. These plasmonic structures are then exploited for dielectric sensing under two configurations: when a thin dielectric film is positioned atop the plasmonic structures and when the analyte surrounds/fills the plasmonic particles/apertures. The complementary sensing performance of both proposed structures is also evaluated, showing the approximate validity of the Babinet principle with sensitivity values of up to ∼650 nm/RIU for thin dielectric sensing.

## 1 Introduction

Plasmonic nano-/micro-structures have been the focus of intense research in recent decades [1–4]. This is due to their ability to confine electromagnetic (EM) fields at sub-wavelength scales [1, 5–9]. The manipulation and control of light–matter interactions at the nano-/micro-scale has been at the core of the field of plasmonics with emphasis on surface plasmon polaritons (SPPs, evanescent surface waves that are excited at the interface between a metal and a dielectric) [10–17] and localized surface plasmon (LSP) resonances excited in plasmonic nanoparticles [18–29] giving rise to localized hotspots [23, 30–35]. The spectral location and properties of the LSP resonances of a plasmonic structure can be controlled by engineering the geometrical parameters of the involved nanoparticles such as size and shape [22, 30, 36–42], the materials used to design them [38, 39, 43], [44], [45] and by combining individual resonances that appear in different nanoparticles via mode hybridization [18, 30], [31], [32, 35, 36, 46], [47], [48].

Classical concepts have also been implemented in plasmonics to understand and design plasmonic nanostructures, such as the study of LSP resonances using conformal mapping (a technique known in antenna engineering [26, 49], [50], [51]). Babinet’s principle for complementary structures is another example of classical techniques that has recently been successfully studied in plasmonic structures [31, 52–57]. This principle states that the diffraction pattern of a set of infinitely thin perfectly opaque particles will be identical to the diffraction pattern of a complementary set of apertures in an infinitely thin, perfectly opaque screen with only a difference in the observed amplitude [52]. Several recent studies have demonstrated that planar plasmonic nanoparticles will have their respective LSP resonances almost at the same spectral location as their complementary versions (nanoapertures) [30, 31, 47, 53], [54], [55], [56]. Small discrepancies are expected [55] in terms of the spectral location of LSP resonances due to, for instance, metals not being perfect conductors at optical frequencies and also the fact that the thickness of plasmonic nanostructures, although small, is not zero as required by the classical Babinet’s principle [58].

One research field that has substantially benefited from the research and innovation of plasmonic nanostructures is the field of sensing (the main focus of this work as it will be detailed below) with some examples including single particle detection [59–61], surface-enhanced Raman spectroscopy [62, 63], gas and ion detection [64, 65], biosensors [66–69] as well as the ability to determine the optical/geometrical properties of local dielectrics (such as the refractive index or their thickness) [70, 71].

In this manuscript, in-depth numerical and experimental studies are carried out to evaluate the sensing performance of plasmonic cylindrical particle-dimers made of gold (from now on just referred to as plasmonic particles) and their complementary version, cylindrical aperture-dimers in a gold screen (from now referred to as plasmonic apertures). The aim of this work is to show how such complementary responses enabled by the Babinet principle in plasmonic nanostructures could be exploited for dielectric sensing. Electron Energy Loss Spectroscopy (EELS) is used to experimentally map the distribution of the LSP modes. These results are compared with numerical simulations via the transmission/reflection spectra and the field distributions (electric and magnetic fields) of their corresponding LSP resonances. After this, as both plasmonic structures confine EM field at nanoscales, a study of their performance when they are used as dielectric sensing devices is presented. Two different configurations are considered for the analyte to be sensed: (i) when an analyte is positioned atop the plasmonic structures and (ii) when the dielectric analyte is surrounding the plasmonic particles or filling the plasmonic apertures. Note that other configurations could also be used such as non-flat [72] or inhomogeneous surfaces of the analyte to account for fabrication errors. Here, however, we focus on the two representative configurations described above with the aim to assess the role of Babinet’s principle in plasmonic sensing devices. It will be shown that the plasmonic apertures exhibit better sensitivity than the plasmonic particles when the analyte is positioned atop the structures while the opposite is true when the analyte is used in the second configuration. In both configurations, sensitivity values in the order of hundreds of nm/RIU are achieved. As the plasmonic sensors present an interchangeable response due to their geometries being complementary, our work may enable the selection of a specific configuration with the best performance depending on the application, such as the type of analyte to be sensed.

## 2 Design and results

### 2.1 Configurations of the plasmonic particles and apertures

**Figure 1:**
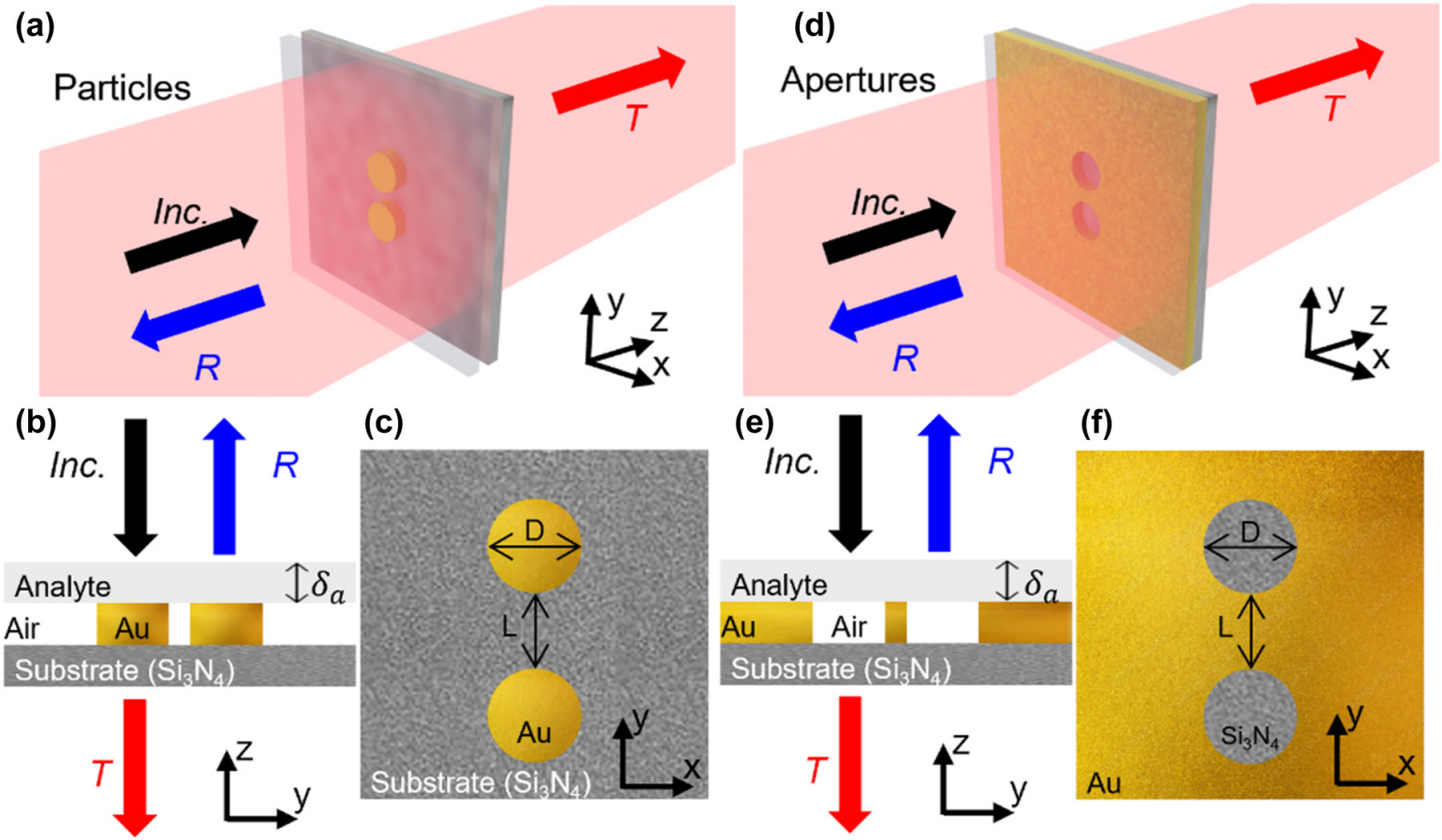
Schematic representation of the complementary plasmonic structures. (a–c) Cylindrical gold (Au) particle-dimers on a silicon nitride (Si_3_N_4_) substrate, (a) perspective, (b) cross-section on the *yz*-plane and (c) *xy*-plane. (d–f) Same as (a–c) but for the complementary plasmonic structure consisting of cylindrical aperture-dimers in an Au film. A dielectric with variable thickness and refractive index is positioned atop these metallic structures which will act as the analyte to be sensed. The two structures are illuminated by an incident plane wave, represented by the black arrow (Inc.), this illumination enables the excitation of LSP resonances. The spectral position of these LSP resonances for each plasmonic structure is determined using the reflection (*R*) and transmission (*T*) coefficients. These parameters are then used to study the sensing capabilities of both plasmonic structures in the presence of the analyte. The parameters*D* and *L* in panel (c and f) represent the diameter and the separation of the cylindrical components, respectively.

Three-dimensional (3D) schematic representations of the two plasmonic structures under study are shown in Figure 1a and d: particles and apertures, respectively. Two-dimensional (2D) cross-sections on the *yz*-plane of these plasmonic structures are also shown in Figure 1b for the plasmonic particles and Figure 1e for the plasmonic apertures, for completeness. As observed, the plasmonic structures are complementary to one another. The plasmonic particles are composed of gold (Au) with a thickness of 30 nm and the complementary structures (plasmonic apertures) are designed using an Au sheet of the same thickness. In so doing, the layer thickness is smaller than the incident wavelength of the illuminating plane wave which will be varied between *λ*_0_ = 0.967 µm to ∼2 µm (310–150 THz), with *λ*_0_ as the wavelength in free space. This is to enable an approximation of Babinet’s principle (which considers infinitely thin metallic particles/screens). The plasmonic particles and apertures are characterized by a diameter *D* and separation between the particles/apertures denoted as *L*. Both structures are placed on top of a silicon nitride (Si_3_N_4_) substrate. In the numerical analysis, the dielectric function of Au is modelled by fitting Johnson and Christy’s experimental data [73]. The substrate, Si_3_N_4_, is modelled using the experimental work from [74]. The structures are illuminated with a plane wave under normal incidence travelling along the *z*-axis. Two orthogonal linear polarizations of the incident illumination are considered, as required to test the validity of Babinet’s principle [30, 55].

Thin dielectric analytes are introduced to the top of the plasmonic structures to evaluate their performance as dielectric sensors. The schematic representation of this setup is also shown in Figure 1. Other sensing scenarios such as the case when the analyte surrounds the plasmonic particles or fills the plasmonic apertures are also studied. Without loss of generality, non-dispersive dielectric analytes are used with real refractive index values (*n*_a_) and variable thickness (*δ*_a_) along the *z*-axis (see Figure 1). Finally, the whole structures are immersed in air (*n*_0_ = 1). For the sake of completeness, the *xy*-plane cross-sections at the top surface of the plasmonic particles and apertures are shown in Figure 1c and f, respectively. As mentioned in the previous section, the size and shape of the plasmonic structures will dictate the spectral location of the LSP resonances. For instance, modifying *D* and/or *L* results in a shift of the LSP resonant wavelength [33, 75], as expected. Based on this, for the numerical studies, these parameters are chosen to be constant (*D* = 200 nm, *L* = 20 nm) in this manuscript. The attention is focused into studying the validity of Babinet’s principle with the proposed designs and their performance when they are implemented as dielectric sensors.

The proposed plasmonic structures are numerically studied using the RF module from the commercial software COMSOL Multiphysics^®^ (see the Methods section below for further details). The full plasmonic structures (particles or apertures on top of a Si_3_N_4_ substrate of thickness 30 nm) are immersed in air (*n*_0_). As shown in Figure 1, the incident plane wave (indicated by the arrow labelled Inc. in Figure 1) interacts with the plasmonic structures to excite LSP resonances. As it will be shown, illuminating the plasmonic particles and apertures with two different linear, orthogonal polarizations will excite different but complementary LSP resonant modes. To determine their spectral position, the reflected (labelled as *R*) and transmitted (labelled as *T*) radiation is retrieved.

### 2.2 Localized surface plasmon modes

**Figure 2:**
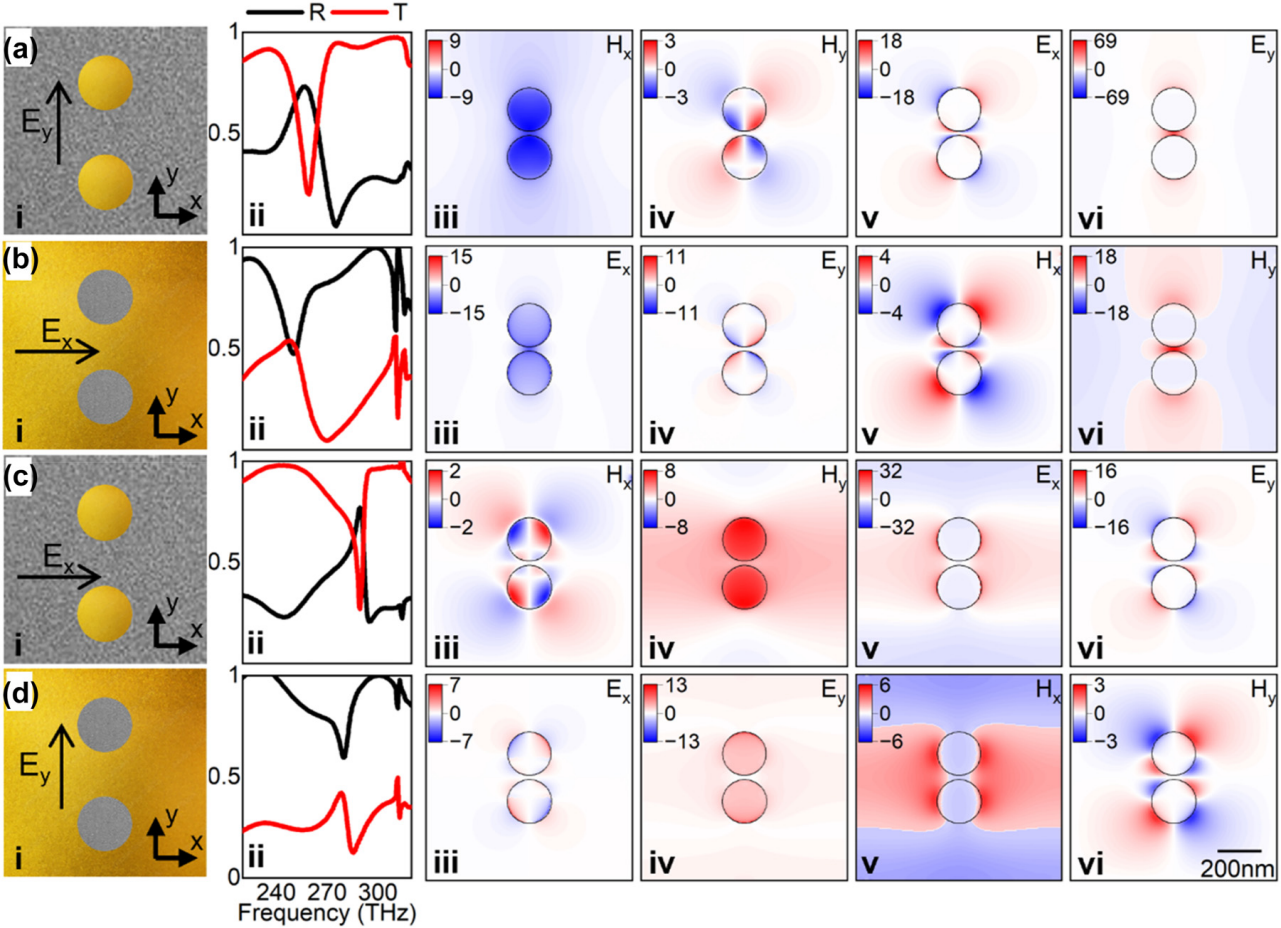
Field distribution of plasmonic particles and apertures. Each row in this figure represents: (a and c) the results considering the plasmonic particles illuminated with a linearly *E*_*y*_ or *E*_*x*_ polarized plane wave, respectively. (b and d) The results considering the plasmonic apertures illuminated with a linearly *E*_*x*_ or *E*_*y*_ polarized plane wave, respectively. The panels along the rows (a and c), corresponding to the plasmonic particles, are organized as follows: (i) two-dimensional (2D) schematic representation on the *xy*-plane of the plasmonic particles showing the direction of the linearly polarized incident plane wave illuminating them, (ii) reflection (black solid line) and transmission (red solid line) spectra, (iii) *H*_*x*_, (iv) *H*_*y*_, (v) *E*_*x*_, and (vi) *E*_*y*_ field enhancements of the LSP resonances: longitudinal dipole bonding (LDB) LSP mode (*f*_0_ ≈ 259 THz/*λ*_0_ ≈ 1.16 µm/*E*_0_ ≈ 1.07 eV) for (a) and a transverse dipole (TD) LSP mode (*f*_0_ ≈ 290 THz/*λ*_0_ ≈ 1.03 µm/*E*_0_ ≈ 1.20 eV) for (c). The field enhancements are calculated as the ratio between the spatial distributions of the full electric or magnetic field and the excitation field. The sign of the field enhancement is then determined from the direction of the specific vectorial component for which the enhancement is evaluated. Similarly, for panels (b and d) corresponding to the plasmonic apertures, the panels along the rows are organized as follows: (i) 2D schematic, (ii) reflection and transmission spectra, (iii) *E*_*x*_, (iv) *E*_*y*_, (v) *H*_*x*_, and (vi) *H*_*y*_ field enhancements of the LSP resonances: complementary longitudinal dipole bonding (cLDB) LSP mode (*f*_0_ ≈ 250 THz/*λ*_0_ ≈ 1.12 µm/*E*_0_ ≈ 1.03 eV) for (b) and a complementary transverse dipole (cTD) LSP mode (*f*_0_ ≈ 280 THz/*λ*_0_ ≈ 1.07 µm/*E*_0_ ≈ 1.16 eV) for (d).

The schematic representations of the plasmonic particles and apertures under study are shown in the first column of Figure 2 [Figure 2a–d(i), top views at the surface of the plasmonic particles/apertures]. In this figure, the linear polarization of the incident signal used to illuminate the plasmonic structures is represented as a horizontal or a vertical arrow depending on the polarization direction of the incident electric field (*E*_*x*_ or *E*_*y*_, respectively). The reflection and transmission for the plasmonic particles and apertures under vertical and horizontal polarization of the incident illumination are shown in the second column of Figure 2 [Figure 2a–d(ii)] as black (*R*) and red lines (*T*). There are minima in the transmission spectra for the plasmonic particles which almost match the minima in the reflection spectra for the plasmonic apertures. These minima are indications of the excitation of LSP resonances in both structures. The existence of these LSP resonances appearing at almost the same location in the spectrum for the transmission coefficient of the plasmonic particles and the reflection of the plasmonic apertures under orthogonal polarization demonstrates that Babinet’s principle is *approximately* but not completely valid in these plasmonic structures. This is an expected result given that the metallic layers are non-infinitely thin and the fact that the metals are not perfect electric conductors at optical frequencies, which are requirements for Babinet’s principle to hold.

The spectral differences (slight shift and overall spectral profile of the resulting LSP resonances) can be quantitatively evaluated by calculating the *Q*-factor as $Q=\frac{\lambda_{\text{res}}}{\text{FWHM}}$ where *λ*_res_ is the wavelength of the LSP resonance and FWHM is its corresponding full-width at half-maximum. The *Q*-factor is useful when evaluating sensors [76, 77]. In the following sections, we use this as a parameter to quantitatively evaluate the plasmonic structures for dielectric analyte sensing. It will be discussed how high *Q*-values are required to detect refractive index changes in the dielectrics used as analytes. As a quantitative example, consider the plasmonic particles illuminated by a plane wave with a vertical *E*_*y*_ polarization as shown in Figure 2a(i). For this scenario, the LSP resonance [minimum transmission, red line in Figure 2a(ii)] is a longitudinal dipole bonding (LDB) LSP mode (obtained at frequency/wavelength/energy *f*_0_ ≈ 259 THz/*λ*_0_ ≈ 1.16 µm/*E*_0_ ≈ 1.07 eV with a *Q*-factor ≈ 22.5). Now, for the complementary plasmonic version, i.e., apertures illuminated by a plane wave with *E*_*x*_ polarization [see Figure 2b(i)] the LSP resonance appears at a frequency of ∼250 THz (*λ*_0_ ≈ 1.20 µm/*E*_0_ ≈ 1.03 eV/*Q*-factor ≈ 16.4) and corresponds to a complementary longitudinal dipole bonding (cLDB) LSP mode [calculated from the minimum in reflection of Figure 2b(ii), black line]. The difference in the spectral positions and *Q*-factors of both resonances is an expected result due to the fact that realistic plasmonic structures are considered (instead of using ideal perfectly conducting metals with almost zero thickness). This produces LSP resonances in the plasmonic particles and complementary plasmonic apertures at similar but not exactly the same spectral positions.

For completeness, let us also consider the scenarios shown in Figure 2c and d(i) for an *E*_*x*_ and *E*_*y*_ polarized incident illumination of the plasmonic particles and apertures, respectively. In these cases, the LSP resonance obtained using the minimum of transmission and reflection for the plasmonic particles and apertures, respectively, are a transverse dipole antibonding (TDA) LSP mode (*f*_0_ ≈ 290 THz/*λ*_0_ ≈ 1.03 µm/*E*_0_ ≈ 1.20 eV/*Q*-factor ≈ 48.1) [Figure 2c(ii)] and a complementary transverse dipole antibonding (cTDA) LSP mode (*f*_0_ ≈ 280 THz/*λ*_0_ ≈ 1.07 µm/*E*_0_ ≈ 1.16 eV/*Q*-factor ≈ 15.1) [Figure 2d(ii)], respectively. Again, this demonstrates how the LSP resonances occur in similar but not the same spectral positions. For the mode labelling see Ref. [30]. Importantly, also note that the *Q*-factor is higher for the plasmonic particles compared to the values obtained with the plasmonic apertures. This will have implications when using these structures as sensors.

Once the spectral location of the excited LSP modes for the plasmonic particles and apertures have been studied, the nature and comparable properties of these plasmonic resonances in terms of their field enhancement distribution (from now on just referred to as field distributions) can be discussed. Here, enhancement is defined as the ratio between the spatial distributions of the full electric or magnetic field and the excitation field with a sign determined from the direction of the specific vectorial component for which the enhancement is evaluated. As demonstrated in [31, 35, 78], the presence of two plasmonic features (cylinders in our case) will generate LSP modes which are the result of hybridization between individual LSP modes excited in each plasmonic particle or aperture. The nature of the hybridized modes is different for both complementary structures (discussed below), but their electric (***E***) and magnetic (***H***) field distributions will be complementary, following Babinet’s principle.

Let us discuss the complementary field distributions for both plasmonic structures (a discussion regarding the distribution of charge mapping the different hybridized LSP modes will be presented in the next sections). The ***E***- and ***H***-field distributions of the plasmonic LSP resonances, calculated at the surface of the plasmonic structures, are shown in Figure 2a–d(iii–vi). For the plasmonic particles (first and third row from Figure 2) the field distributions are calculated for the LDB and TDA LSP modes, respectively, i.e., at the frequency of minimum transmission. For the plasmonic apertures (second and fourth rows from Figure 2 corresponding to the cLDB and cTDA LSP modes, respectively) the LSP resonances are calculated at the minimum of reflection. With this configuration, let us first compare the results shown in Figure 2a and b. The horizontal (*H*_*x*_) [Figure 2a(iii)] and vertical (*H*_*y*_) [Figure 2a(iv)] components of the ***H***-field for the plasmonic particles under vertical polarization of the incident plane wave resembles the horizontal (*E*_*x*_) [Figure 2b(iii)] and vertical (*E*_*y*_) [Figure 2b(iv)] components of the ***E***-field distribution of the plasmonic apertures, respectively. This is also evident when comparing the *E*_*x*_ [Figure 2a(v)] and *E*_*y*_ [Figure 2a(vi)] field distributions of the LSP resonance for the plasmonic particles with the *H*_*x*_ [Figure 2b(v)] and *H*_*y*_ [Figure 2b(vi)] field distributions of the plasmonic apertures. Finally, the same complementarity is also observed for the field distributions shown in Figure 2c and d, as it should happen when considering Babinet’s principle [79]. Quantitatively, note that from Figure 2, the components for the ***E***- and ***H***-fields have different magnitudes, in agreement with [55].

### 2.3 Experimental comparison

The plasmonic particles and apertures were fabricated using the process detailed in the Methods section. To characterize the morphology and optical properties (i.e., LSP modes) of the dimers, the sample was analyzed by scanning transmission electron microscopy (STEM) in combination with electron energy loss spectroscopy (EELS) in a transmission electron microscope FEI Titan in monochromated scanning regime (see Methods section for further details).

**Figure 3:**
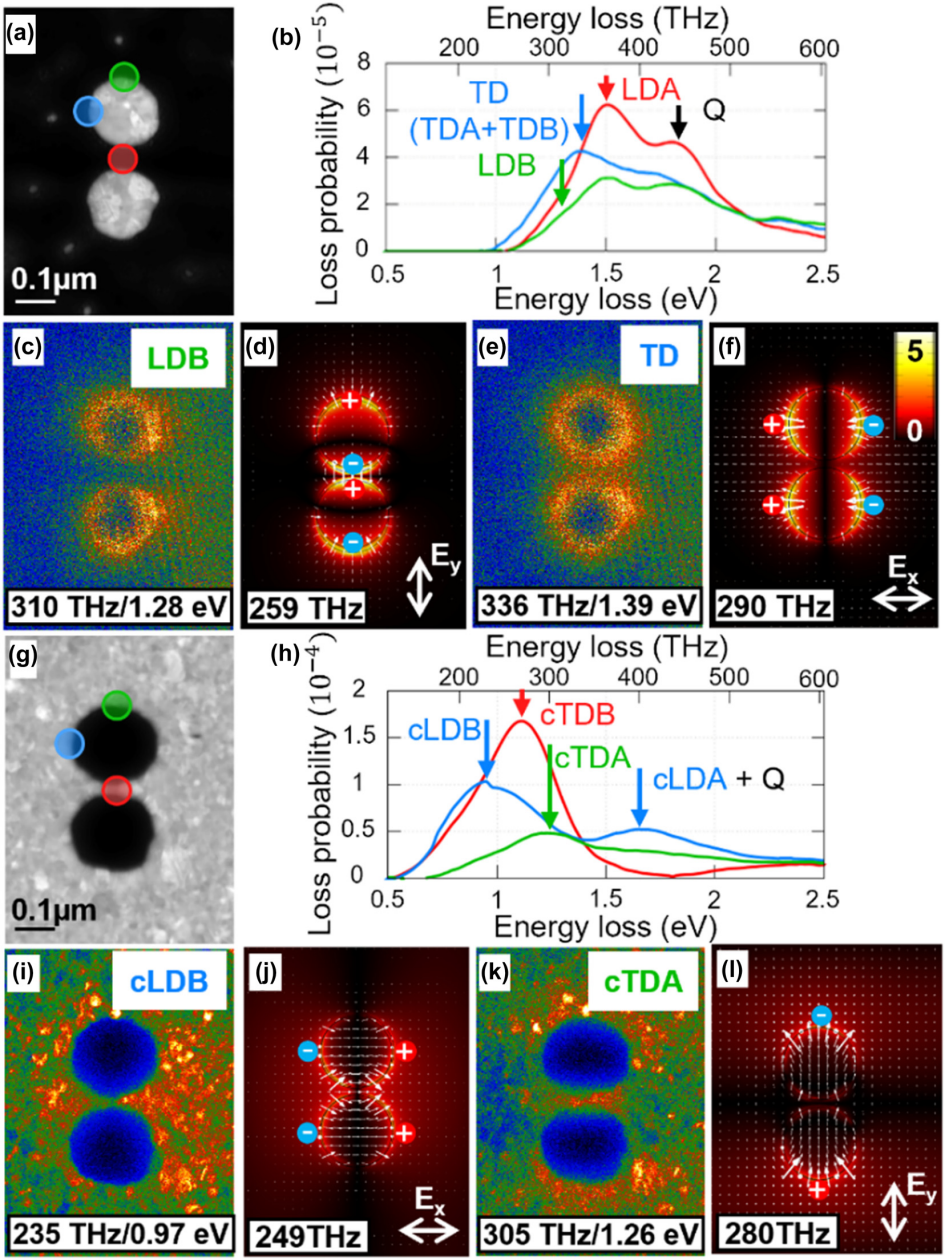
STEM characterization of the plasmonic particle and aperture dimers. (a and g) ADF-STEM images of a particle and aperture dimer, respectively. For the plasmonic particles, the real dimensions are: diameters *D*_t_ = 172 nm (top particle) and *D*_b_ = 172 nm (bottom particle) and the gap *L* = 50 nm, while for the plasmonic apertures, the real dimensions are *D*_t_ = 210 nm (top aperture) and *D*_b_ = 200 nm (bottom aperture) and *L* = 20 nm. (b and h) Loss probability (per the spectral range of 0.01 eV) for the plasmonic particles and apertures, respectively, measured at positions marked in panels (a and g). (c and e) Spatial maps of the loss probability for the plasmonic particles shown in (a) for a frequency of the LDB and TD modes, respectively. (d and f) Numerical results of the |*E*_*z*_| field distribution on the surface of the plasmonic dimers (*D*_t_ = *D*_b_ = *D* = 200 nm, *L* = 20 nm) when they are illuminated by a plane wave with *E*_*y*_ polarization at a frequency of the LDB mode and *E*_*x*_ polarization at a frequency of TDA mode, respectively, along with the corresponding electric field lines. In these panels, the “(+)” (red) and “(−)” (blue) symbols represent the surface charge distributions. (i and k) Same as (c and e) but for the plasmonic apertures at the frequency of the cLDB and cTDA modes, respectively. (j and l) Same as (d and f) when considering the complementary apertures using an *E*_*x*_ polarized incident plane wave at the frequency of cLDB mode and *E*_*y*_ polarization at a frequency of cTDA mode, respectively.

Annular dark-field (ADF) STEM images of the fabricated plasmonic particles and apertures are shown in Figure 3a and g, respectively. The real dimensions of the structures determined from ADF-STEM images slightly differ from the targeted ones (the cylinder diameter of 200 nm and the gap between the dimers of 20 nm). For the plasmonic particles (Figure 3a) a cylinder diameter of 172 nm (both particles) and a gap of 50 nm were obtained (due to fabrication limitations), while the plasmonic apertures, shown in Figure 3g, have the diameters of 210 nm (top aperture) and 200 nm (bottom aperture) and a gap of 20 nm.

The LSP modes supported by plasmonic dimers are characterized by EELS. A beam of probing electrons is transmitted through the sample and an energy spectrum of inelastically scattered electrons is recorded. The spectrum is contributed also by the electrons that excited an LSP mode in a plasmonic dimer, and in turn, decreased their energy by the energy of the LSP mode. The excitation of an LSP mode can be described within the framework of classical electrodynamics where the EM field induced by the LSP acts back on the electron [80, 81], resulting in the loss probability [55]:

(1) $$\Gamma_{\text{EELS}}\left(\omega \right)=\frac{e}{\pi \hbar \omega }\int dtRe\left\{e^{-i\omega t}v\cdot E_{ind}\left[r_{e}\left(t\right),\omega \right]\right\}$$

where *e* is the charge of an electron, *ω* the working angular frequency, ℏ is the reduced Planck constant, *t* is time and $E_{ind}\left[r_{e}\left(t\right),\omega \right]$ is the induced electric field from an electron moving with a velocity ***v*** at the electron position $r_{e}\left(t\right)$ . Now, if one considers an electron moving along the *z*-axis (perpendicular to the plasmonic particles or apertures) only the out-of-plane component of the electric field (*E*_*z*_) of the plasmonic structures will interact with the probing electrons, making EELS insensitive to in-plane *E*-fields (*E*_*x*_ and *E*_*y*_). The LSP-related losses are proportional to the *E*_*z*_ of the excited LSP resonances. The experimental EEL spectrum is further contributed by material-related bulk losses which are proportional to the thickness of the metallic structure. To isolate the LSP-related losses a subtraction of the zero-loss peak and background was performed, and the spectra were normalized.

**Figure 4:**
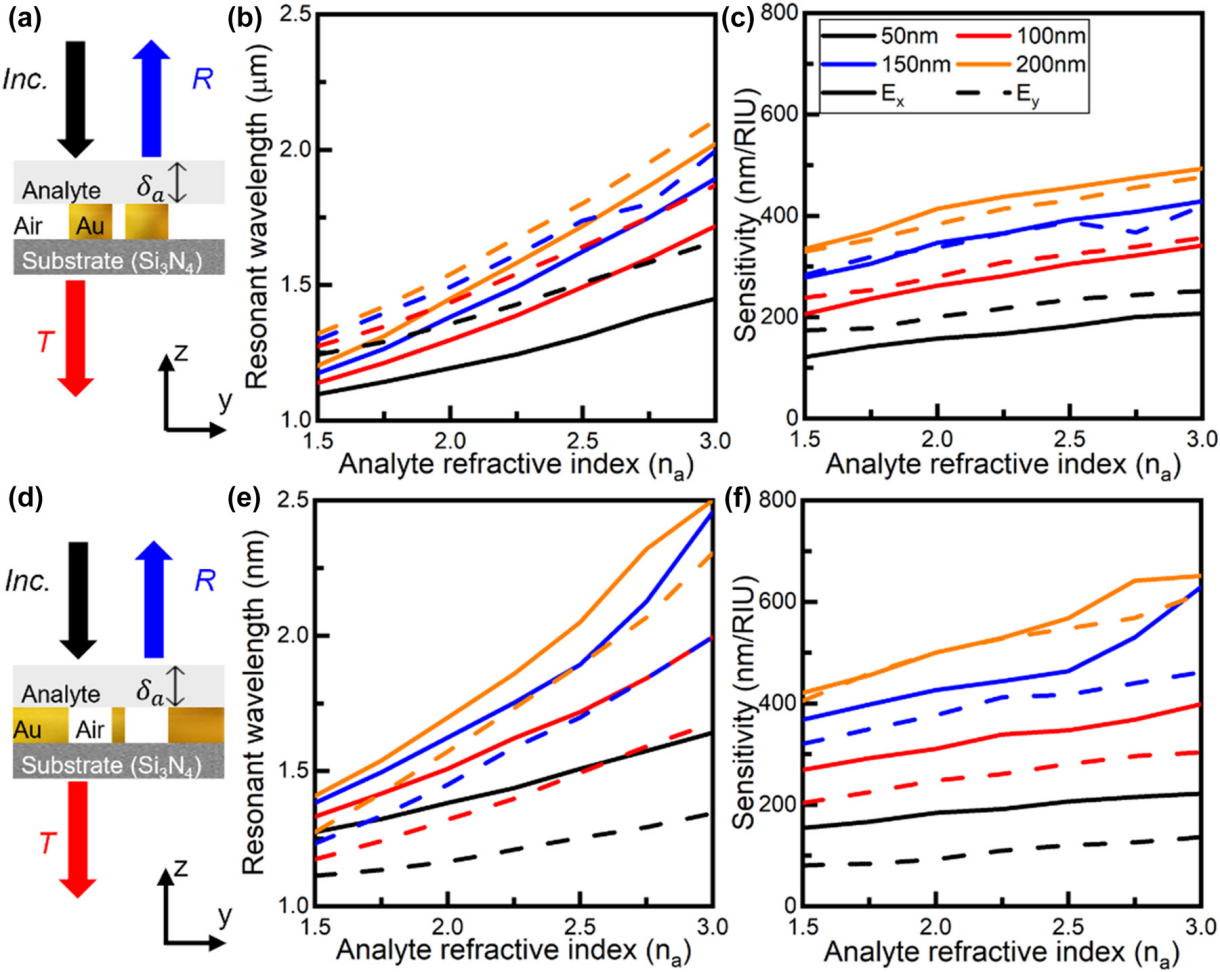
Resonant wavelength and sensitivity of the plasmonic structures with an analyte positioned atop. 2D Schematic representation of the cross-section on the *yz*-plane for (a) plasmonic particles and (d) apertures used to determine changes of a nearby thin film analyte. Resonant wavelengths of the LSP modes produced by the (b) plasmonic particles shown in (a), and (e) the plasmonic apertures from (d) when *n*_a_ is changed from 1.5 to 3 in steps of 0.25 considering an incident plane wave with *E*_*x*_ (solid line) and *E*_*y*_ (dashed line) polarization. The thickness of the analyte is chosen to be: *δ*_a_ = 50 nm (black), *δ*_a_ = 100 nm (red), *δ*_a_ = 150 nm (blue) and *δ*_a_ = 200 nm (orange). Sensitivity of the (c) plasmonic particles and (f) apertures when illuminated by a plane wave with *E*_*x*_ (solid) and *E*_*y*_ (dashed) polarization, for analyte thicknesses of *δ*_a_ = 50 nm (black), *δ*_a_ = 100 nm (red), *δ*_a_ = 150 nm (blue) and *δ*_a_ = 200 nm (orange).

Experimental EEL spectra are shown in Figure 3b for the plasmonic particles and in Figure 3h for the plasmonic apertures. The identification of the LSP modes in the plasmonic particles spectra follows the procedure described in Ref. [82] and the interpretation of the plasmonic apertures spectra was carried out by using the electric–magnetic Babinet complementarity [30]. The spectral positions of all hybridized dipole modes [83] [i.e., LDB, transverse dipole bonding (TDB), transverse dipole anti-bonding (TDA), and longitudinal dipole antibonding (LDA)] supported by the plasmonic particles and Babinet-complementary modes supported by the plasmonic apertures (with abbreviations prefixed with c, i.e., cLDB, cTDB, cTDA, cLDA), are marked with arrows in Figure 3b and h. Here, the label TD is used for unresolved TDB and TDA modes supported by the plasmonic particles and *Q* for the contribution of the quadrupole mode (disregarding its hybridization). Further multipole analysis could be done to explore higher-order modes such as in [82, 84, 85]. In the following, the attention is focused on bright LSP modes with non-zero dipole electric moment as they couple to the EM plane wave and are manifested in the transmission and reflection spectra of Figure 2. They include LDB and TDA modes of the plasmonic particles and cLDB and cTDA modes of the plasmonic apertures. The other dipole LSP modes are dark [20, 22]. These modes fall outside of the scope of this work as we exploit bright modes for dielectric sensing in the following sections. The spectral positions of the bright LSP modes are shown in Table 1. As observed, they correspond well to the spectral positions obtained from the calculated optical spectra and they also approximately follow Babinet’s principle. The differences between the numerical and experimental location of the LSP resonances may be attributed to fabrication tolerances introducing differences in the dimensions between the fabricated and numerically simulated plasmonic structures. These may include, for instance, the fabricated plasmonic dimers being not completely cylindrical or the Si_3_N_4_ substrate not being perfectly 30 nm thick, among others. A study of the influence of these potential errors is shown in the Supplementary Materials. Moreover, as shown in Table 1, somewhat larger mode energies observed for the plasmonic particles can be attributed to the cylinder diameter being smaller than designed (by about 14 %). Based on the approximate linear dispersion relation between the plasmon resonance energy and reciprocal diameter of the disc antenna which holds for gold plasmonic antennas (see e.g. [30] and Figure 4 therein) even better agreement can be obtained by qualitatively correcting the spectral position by about 14 % (and for the aperture dimer by about 2.5 % upwards). It is also of note that EELS and transmission/reflection spectra are near- and far-field based calculations, respectively, which may be a potential source of deviations for the LSP resonant wavelengths between simulations and EELS [86]. Figure 3c, e, i and k show the spatial maps of the experimental loss probability at the energy of the LDB and TDA modes (Figure 3c and e, the plasmonic particles) and cLDB and cTDA modes (Figure 3i and k, the plasmonic aperture). To reduce the noise, the loss probability maps are integrated over a spectral range of 0.1 eV centered on the mode energy.

**Table 1:** Spectral positions of the bright dipole LSP modes supported by the plasmonic particles and the plasmonic apertures.

Mode	Experimental	Corrected	Calculated		
	*f*_0_ (THz)	*f*_0_ (THz)	*f*_0_ (THz)	*E*_0_ (eV)	*λ*_0_ (μm)
**Particles**					
LDB	310	267	259	1.07	1.16
TDA	336	289	290	1.20	1.03
**Apertures**					
cLDB	235	241	250	1.03	1.12
cTDA	305	312	280	1.16	1.07

Experimental EELS maps (Figure 3c, e, i and k) are compared with numerically calculated out-of-plane electric field $\left(\left|E_{z}\right|\right)$ distributions, shown in Figure 3d, f, j and l. The experimental loss probability maps shall qualitatively agree with the calculated |*E*_*z*_| distribution, as predicted by Eq. (1). This is indeed confirmed for the plasmonic apertures (Figure 3i–l). For the plasmonic particles, the larger gap between the cylinders (50 nm) in the fabricated case compared to the numerical simulation (20 nm) resulted in a strong spectral overlap of all hybridized dipole modes due to their smaller energy difference. This makes the visual comparison more challenging but qualitative agreement with numerical simulations is still present (Figure 3c–f). The red circles with “(+)” symbols and blue circles with “(−)” symbols in Figure 3d, f, j and l schematically show the accumulation of charges of each LSP mode. The distribution of charges together with the direction of the electric field allows us to identify the LSP modes as longitudinal/transverse and bonding/antibonding, as detailed in Ref. [30].

### 2.4 Thin film sensing

Let us now study the potential of both complementary plasmonic structures to sense variations of a nearby dielectric thin film. The schematic representations of the plasmonic particles and apertures explored in the previous sections with a thin dielectric film positioned atop (which will act as the analyte in our case) is shown in Figure 4a and d, respectively. The refractive index, *n*_a_, and thickness, *δ*_a_, of the analyte is changed to shift the spectral position of the LSP resonances. This spectral shift is then mapped by recording the transmission and reflection spectra of the plasmonic structures (particles and apertures, respectively). This shift in the spectral position of the LSP resonances is shown in Figure 4b and e for the plasmonic particles and apertures, respectively. Here, the plasmonic structures are illuminated with a plane wave under *E*_*x*_ (solid lines) and *E*_*y*_ (dashed lines) polarization. The thickness of the analyte is then considered to be *δ*_a_ = 50 nm (black), 100 nm (red), 150 nm (blue) and 200 nm (orange) and its refractive index (*n*_a_) is varied from 1.5 to 3 in steps of 0.25. As it can be seen in Figure 4b and e, the change of the resonant wavelength of the LSP mode for the plasmonic particles is more prominent when using *E*_*y*_ polarization (dashed lines) while the spectral shift when using the plasmonic apertures is larger when illuminated by an orthogonally polarized plane wave (*E*_*x*_ polarization, solid lines). These are expected results due to the interaction between the analyte and the field distribution of the corresponding LSP modes for each plasmonic structure which produce high field concentrations near the plasmonic structures.

To further evaluate the sensing features of the plasmonic structures, their sensitivity can also be calculated. The sensitivity is defined as the ratio between the change of the wavelength of the LSP resonances and the refractive index variation of the analyte, $S=\frac{\Delta \lambda }{\Delta n_{\text{a}}}$ , $\left[\frac{\text{nm}}{\text{RIU}}\right]$ , with RIU as refractive index unit [87–89]. The calculated results are shown in Figure 4c and f for the plasmonic particles and apertures, respectively. As observed, the sensitivity of both plasmonic structures increases as *δ*_a_ and *n*_a_ of the analyte increases, as expected, in line with the results discussed in Figure 4b and e. Quantitatively, the sensitivity is increased from ∼100 nm/RIU for both the plasmonic particles and apertures (when *δ*_a_ = 50 nm and *n*_a_ = 1.5) up to ∼450 nm/RIU and ∼650 nm/RIU for the plasmonic particles and apertures, respectively (when *δ*_a_ = 200 nm and *n*_a_ = 3). It is important to note that when the thickness of the analyte on top of the plasmonic particles is *δ*_a_ < 100 nm there is a clear difference between sensitivity values for the two orthogonal polarizations of the incident illumination, with larger sensitivities when using the *E*_*y*_ polarization. For the plasmonic apertures, the sensitivity values are generally higher when illuminated by a plane wave with *E*_*x*_ polarization (as discussed before in terms of the wavelength shift). From these results with both plasmonic structures, their increased sensitivity depending on the polarization of the illumination is due to the excitation of a hotspot in the field distribution: ***E***-field hotspot between the plasmonic particles and a complementary ***H***-field hotspot between the plasmonic apertures. Moreover, as the analyte touches the plasmonic apertures on all its surface, compared to the plasmonic particles where only the top surface of the particles is in contact to the analyte (see Figure 4a and d), these field hotspots will cause a more significant interaction of LSP modes with the analyte for the plasmonic apertures, increasing the shift in the resonant wavelength compared to the plasmonic particles (Figure 4b and e) making them more sensitive to changes of the analyte as shown in Figure 4c and f. The values of sensitivity shown here are in line with values found in the literature for similar structures [87, 90, 91].

Finally, it is important to note that when using thicker dielectrics (*δ*_a_ > 100 nm) for both plasmonic particles and apertures, the sensitivity values obtained with both polarizations of the illuminating signal are similar (i.e. almost independent on the polarization). We hypothesize that the change in sensitivity observed with thick dielectrics may be mainly influenced by the multiple reflections within the dielectric rather than the interaction with the LSP resonance of the plasmonic particles and apertures, producing similar values of sensitivities regardless of the polarization of the incident plane wave. A study comparing the sensitivity of the plasmonic particles and apertures when using a smaller amount of a dielectric analyte (size 200 nm × 100 nm × 50 nm) is shown in the Supplementary Materials. These results demonstrate that LSP modes with field hotspots produce higher sensitivity values due to their enhanced interaction with the small dielectric analyte, as expected.

### 2.5 Complementary dielectric sensing: an alternative sensing approach

**Figure 5:**
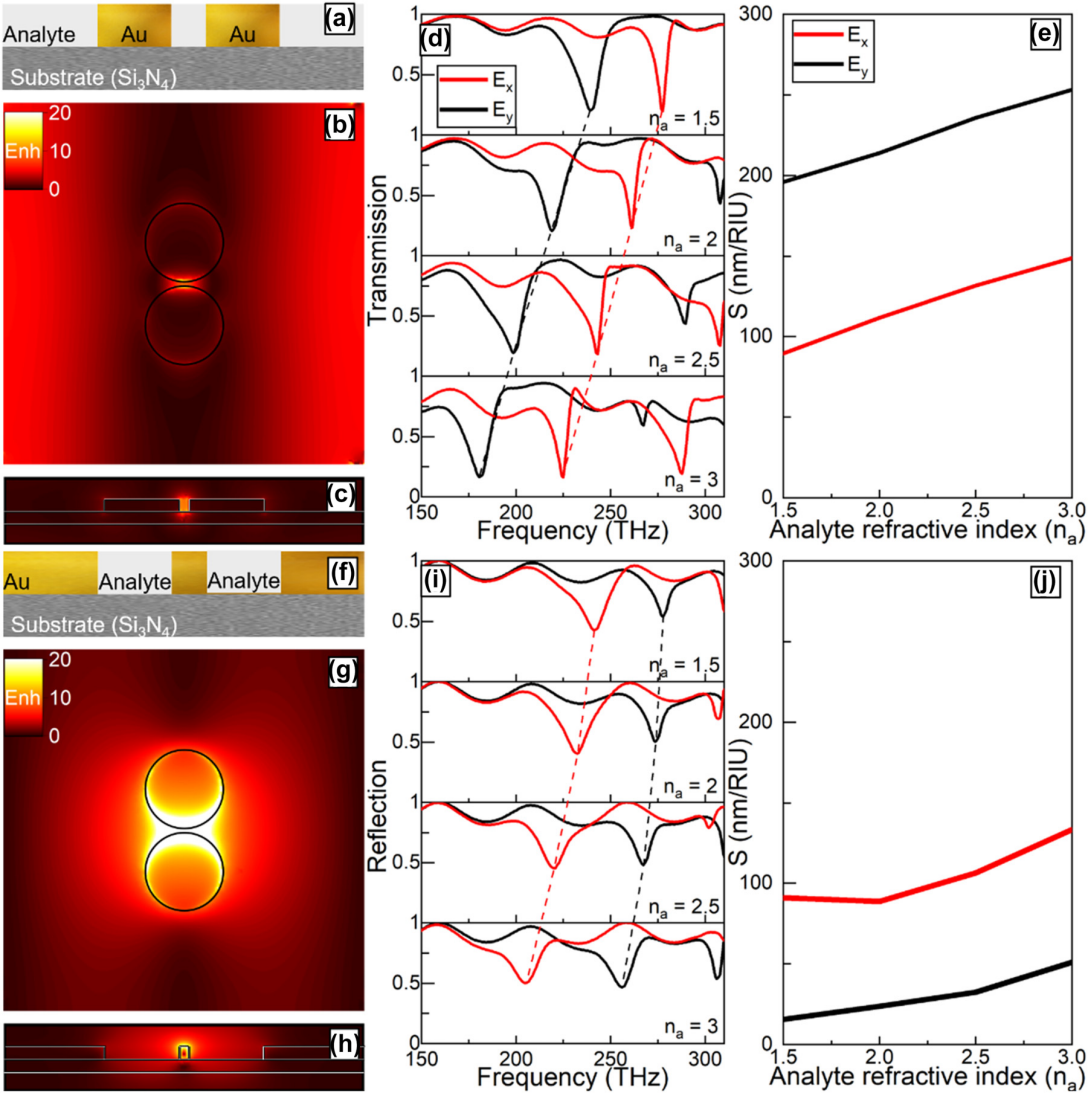
The plasmonic structures sensing an analyte as a part of the structures. 2D cross-section of (a) plasmonic particles and (f) apertures surrounded and filled by a dielectric analyte, respectively. Power enhancement on the (b and g) *xy*-plane and (c and h) *yz*-plane for the plasmonic particles and apertures shown in (a and f), respectively, considering an analyte (*n*_a_ = 1.5). The structures are illuminated with a plane wave under *E*_*y*_ or *E*_*x*_ polarization, respectively. Note that the results in (g and h) have been saturated to use the same scale as (b and c) to better observe the difference in power enhancement. (d) Transmission and (i) reflection spectra produced when illuminating the structures shown in (a and f), respectively, with an *E*_*x*_ (red) and *E*_*y*_ (black) polarized plane wave when *n*_a_ is changed from 1.5 to 3 in steps of 0.5 (panels from top to bottom). Dashed lines going through the minima of the spectra for each value of *n*_a_ have been added to visualise the shift in the spectral location of the LSP resonance. Sensitivity of the (e) plasmonic particles and (j) apertures when they are illuminated by a plane wave with orthogonal *E*_*x*_ (red) and *E*_*y*_ (black) polarizations as *n*_a_ is changed from 1.5 to 3 in steps of 0.25.

In the previous section, the performance of the proposed plasmonic particles and apertures was studied when working as thin dielectric sensors. Importantly, when the analyte is on top of the plasmonic structures, fabrication may be a challenge as the analyte may need to be flat on both sides [72]. For completeness, and to evaluate the response of the plasmonic structures using alternative sensing configurations, a final study is carried out to investigate the sensitivity of the plasmonic particles and apertures when, instead of using a thin film positioned atop the metallic structures, the dielectric analyte is used as part of the complementary plasmonic structures. A 2D schematic representation of the cross-section of the cylindrical plasmonic particles is shown in Figure 5a. It can be seen that the particles are immersed within the dielectric analyte (rather than air as in the previous sections) with both (analyte and particle) having the same thickness. Similarly, to study a complementary sensing approach, the plasmonic apertures are then filled with the dielectric analyte as it is schematically represented in Figure 5f. With this configuration, the numerical results of the power enhancement on the *xy*- and *zy*-planes for the plasmonic particles in a dielectric analyte (*n*_a_ = 1.5) illuminated by an *E*_*y*_ polarized plane wave with a frequency of ∼239 THz (*λ*_0_ ≈ 1.25 µm/*E*_0_ ≈ 0.99 eV) is shown in Figure 5b and c, respectively. A power enhancement (defined as the ratio between the spatial power distribution with and without using the plasmonic structures) of ∼20 is obtained at the gap of the plasmonic particle structure, showing a field hotspot. The power enhancement of the complementary plasmonic structure, double cylindrical dielectrics in a metallic sheet illuminated by an orthogonally polarized plane wave at ∼241 THz (*λ*_0_ ≈ 1.24 µm/*E*_0_ ≈ 1.00 eV) on the *xy*- and *zy*-plane is shown in Figure 5g and h, respectively. Here, a field hotspot with a power enhancement of ∼60 between the plasmonic apertures is observed as the LSPs are tightly bound to the Au bridge between the two apertures. As expected by Babinet’s principle, both complementary structures have similar power enhancement distributions although the magnitude of the power enhancements differs, as discussed in the previous sections.

The effect of changing the refractive index *n*_a_ of the dielectric analyte on the transmitted and reflected signals for the plasmonic particles and apertures, respectively, is shown in Figure 5d and i. Here, the resonant frequency (minima of the spectra, dashed lines have been added to guide the eye) of the LSPs modes is red shifted as *n*_a_ increases, as expected from Figure 4b and e. The sensitivity values of the plasmonic particles and apertures are shown in Figure 5e and j, for both orthogonal plane wave polarizations: *E*_*x*_ (red) and *E*_*y*_ (black). The sensitivities of the plasmonic particles, Figure 5a and e, as *n*_a_ changes from 1.5 to 3 is found to vary from 196 to 253 nm/RIU under *E*_*y*_ illumination and from 90 to 150 nm/RIU when illuminated by an *E*_*x*_ polarized plane wave. On the other hand, the sensitivity of the filled plasmonic apertures, shown in Figure 5f and j, ranges from 91 to 133 nm/RIU and from 15 nm/RIU to 50 nm/RIU when using a plane wave with *E*_*x*_ and *E*_*y*_ polarization, respectively. Similar to the thin dielectric analyte atop the structures studied in the previous section, having the analyte as part of the plasmonic particles and apertures also increases the sensitivity as the refractive index increases. However, by comparing the results from Figures 4c and f and 5e and j, it can be seen that using the analyte as a part of the plasmonic particles and apertures structures (Figure 5e and j) reduces the volume of analyte required to achieve sensitivity values of the same order of magnitude compared to those shown in Figure 4c and f. From Figure 5e and j, the plasmonic particles and apertures illuminated by an *E*_*y*_ or *E*_*x*_ polarized plane wave, respectively, achieve sensitivities of up to ∼250 nm/RIU and ∼150 nm/RIU (*n*_a_ = 3), respectively, with analytes of thickness *δ*_a_ = 30 nm. These results are of the same order of magnitude as the sensitivities achieved, ∼200 nm/RIU, when using a thin dielectric of *δ*_a_ = 50 nm on top of the plasmonic structures (Figure 4c and f). By comparing the results from Figure 4 and those from Figure 5 one can also notice the following: for the plasmonic particles the sensing performance is improved when the particles are immersed within the analyte (Figure 5e) compared to the case when the *δ*_a_ = 50 nm analyte is placed atop (Figure 4c). The opposite occurs with the plasmonic apertures where the sensitivity is improved when the *δ*_a_ = 50 nm analyte is placed atop the apertures (Figure 4f) compared to when the analyte is filling the apertures (Figure 5j). This demonstrates that both plasmonic structures have complementary performances which may be applied for dielectrics sensing with a configuration that could be chosen depending on the type of dielectric to be sensed. In the Supplementary Materials, a discussion of the sensitivity normalized to the volume of the analyte is presented. These results may find applications in dielectric sensing and biosensing, among others.

## 3 Conclusions

In this work, Babinet’s principle has been studied in the realm of plasmonics to develop sensors exploiting LSP resonances in complementary metal-dielectric structures. First, the Babinet’s principle in plasmonics was numerically analyzed by comparing the field distribution of the excited LSP resonances that exist in complementary cylindrical metallic particle-dimers and aperture-dimers in a metallic film. To further study them, these results were compared to experimentally fabricate plasmonic structures with the LSPs resonances mapped by EELS showing good agreement between them in terms of the spectral positions of the excited LSP modes and their charge distribution. These structures were then used as dielectric sensors demonstrating how the LSP resonances can be shifted when using nearby dielectrics under two configurations: thin dielectric atop the plasmonic structures, or plasmonic particles/apertures immersed/filled with a thin dielectric. Complementary sensing performance was also demonstrated showing sensitivity values in the order of several hundreds of nm/RIU for thin dielectrics of thicknesses as small as 30 nm.

## 4 Methods

### 4.1 Numerical simulations

The Au and Si_3_N_4_ square structures in Figure 1 have dimensions 950 × 950 nm with periodic boundary conditions on the top, left, bottom and right boundaries (i.e., *x*-, *y*-boundaries). In so doing the structures are infinitely repeated along the *x*- and *y*-axes. Note that the large lateral size of the simulation domain (950 × 950 nm) has been chosen to minimize lattice resonances. An extremely fine mesh was then applied with a maximum and minimum element size of 2.19 × 10^−7^ m and 9.38 × 10^−9^ m, respectively. Moreover, to further improve the mesh of the plasmonic structure, two extra automatic refinements were applied to the layer containing the plasmonic features (particles or apertures). Two ports (one at the front and one at the back of the structures) are implemented to apply the incident plane wave and to record the transmitted signal, respectively. These ports are each placed at a distance 3000 nm, along the *z*-direction, away from the input (metal)/output (substrate) surfaces of the plasmonic structures. As explained before, the incident plane wave has *E*_*x*_ or *E*_*y*_ polarization (transverse or parallel to the long axis of the plasmonic structures, respectively, see Figure 1). The magnitude of the transmitted and reflected signals as a function of frequency is calculated by recording the scattering parameters (*S*-parameters) at the input/output ports as defined by COMSOL Multiphysics^®^. The reflection and transmission spectra are then used for each of the plane wave illuminated plasmonic structures to determine the spectral position of each LSP resonance.

### 4.2 Fabrication

Both the plasmonic particles and apertures were prepared using a standard focused ion beam (FIB) lithography process [92] which produces high-quality polycrystalline plasmonic antennas fully equivalent to monocrystalline ones [93]. First, a 30 nm-thick gold layer was deposited by magnetron sputtering on a standard silicon nitride membrane for transmission electron microscopy (TEM) with lateral dimensions of 250 × 250 µm^2^ and a thickness of 30 nm. Second, the dimers were fabricated by FIB milling (using Ga+ ions at 30 keV) of the gold layer in a dual beam microscopy system FEI Helios. The particles were set in the centre of a gold-free rectangular area with the size of 3 × 2 µm^2^ to prevent any undesired interaction with the surrounding gold frame.

### 4.3 Characterization

The parameters of the transmission electron microscope FEI Titan used for the EELS measurements were set as follows: the electron energy of 300 keV, with the beam current around 100 pA, the convergence semi-angle of 10 mrad, and the collection semi-angle of 56 mrad. The full width at half maximum of the zero-loss peak read 0.14 eV, and this value roughly represents the spectral accuracy of EELS. Note that prior to the STEM-EELS measurements, the sample was cleaned in argon–oxygen plasma for 20 s to prevent the sample from carbon contamination evolution during the measurement.

## Supplementary Material

Supplementary Material Details
