# Supplementary material for: Plasmonic sensing using Babinet’s principle
Source: Nanophotonics. 2023 Sep 27;12(20):3895–909. doi: 10.1515/nanoph-2023-0317 (PMC11501113; doi:10.1515/nanoph-2023-0317)
Supplement: Supplementary file 1 — Supplementary Material Details [file j_nanoph-2023-0317_suppl_001.pdf]

Supplementary information

Plasmonic sensing using Babinet's principle

Joseph Arnold Riley[1,2,#], *Michal Horák*[3,4#], *Vlastimil Křápek*[3,4,*], *Noel Healy*[1], *and Victor Pacheco-Peña*[1,*]

[1]*School of Mathematics, Statistics and Physics, Newcastle University, Newcastle Upon Tyne, NE1 7RU, United Kingdom*
[2] *School of Engineering, Newcastle University, Newcastle Upon Tyne, NE1 7RU, United Kingdom*
[3]*Central European Institute of Technology, Brno University of Technology, Purkyňova 123, 612 00, Brno, Czech Republic*
[4]*Institute of Physical Engineering, Brno University of Technology, Technická 2, 616 69, Brno, Czech Republic*

1. **Influence of potential fabrication errors of the substrate**
2. **Effect of analyte volume on the sensitivity of the plasmonic dimers**
3. **Sensitivity of plasmonic particles and apertures for small dielectric analytes**

[#] *These authors contributed equally to this work*
[*]*emails: victor.pacheco-pena@newcastle.ac.uk, vlastimil.krapek@ceitec.vutbr.cz*

1. Influence of potential fabrication errors of the substrate

Here we consider the plasmonic particles and apertures discussed in Fig. 3 from the main text with the simulated structures having a reduced substrate thickness (from 30 nm to 20 nm). The LSP resonant frequency of the simulated and experimentally realized plasmonic dimers can be seen in Table S1. As observed, by decreasing the thickness of the substrate of the plasmonic particles and apertures, there is an increase in the LSP resonant frequency closer to the values determined from the loss probabilities of the experimentally realized plasmonic structures. Therefore, fabrication tolerances causing nanometer variations in the desired dimensions of the structures may cause differences between the LSP resonance frequencies of the simulated and fabricated plasmonic particles and apertures.

Table S1. LSP resonant frequencies of simulated and experimentally realized plasmonic dimers with varying substrate thickness.

	Experimental	Simulation substrate thickness	
		20 nm	30 nm
Planewave polarization	f_0(THz)	f_0(THz)	f_0(THz)
**Particle**			
E_x	336	290	299
E_y	310	259	269
**Aperture**			
E_x	235	249	259
E_y	305	280	285

2. Effect of analyte volume on the sensitivity of the plasmonic dimers

Here we consider the sensitivity of the plasmonic particles and apertures as discussed in Fig. 4 and Fig. 5 of the main text relative to the different volumes of the dielectric analyte. When the analyte is positioned atop the whole plasmonic structure (950 × 950 nm) as is the case in Fig. 4 from the main text, the thin dielectric has a volume ($V = width \times length \times \delta_a$) depending on the thickness of the analyte (δ_a = 50 nm, 100 nm, 150 nm, 200 nm) of $V_{50nm} = 4.5125 \times 10^8\ nm^3$, $V_{100nm} = 9.025 \times 10^8\ nm^3$, $V_{150nm} = 1.35375 \times 10^8\ nm^3$ and $V_{200nm} = 1.805 \times 10^9\ nm^3$, respectively. Whereas, when the analyte was used to surround/fill the plasmonic particles/apertures (Fig. 5 of the main text) the volume is $V_{particles} = (x \times y \times \delta_a) - 2 \times (\pi r^2 \delta_a)$ / $V_{apertures} = 2 \times (\pi r^2 \delta_a)$, with δ_a = 30 nm. From this, the analyte has a reduced volume of $2.519 \times 10^7\ nm^3$ and $1.885 \times 10^6\ nm^3$, for the plasmonic particle and aperture configurations, respectively. Then, by using the sensitivities of the plasmonic particles, shown in Fig. 4c and Fig. 5e of the main text, and the plasmonic apertures, shown in Figure 4f and Figure 5j, the volume sensitivity, $S_V = \frac{S}{V}, [\frac{nm}{RIU\ nm^3}]$, can be calculated. The results of the normalized sensitivity of the plasmonic particles and apertures by the volume of the thin film analyte are shown in Fig. S1a,b respectively. As observed, S_V decreases as δ_a (and therefore the volume) increases. This is an expected result because, for instance, the volume of the analyte when δ_a = 200 nm is ×4 greater than when δ_a = 50 nm but the sensitivity (without being normalized to the volume) when using δ_a = 200 nm is only ~×2 larger than when δ_a = 50 nm. Therefore, this means that after normalization with the volume of the analyte, the values of the normalized sensitivity will be more favorable for smaller volumes. In Fig. S1a, the results of the sensing performance for plasmonic particles are shown to provide better values of S_V when illuminated by an E_y polarized plane wave while the E_x polarized plane wave had better values for the plasmonic apertures, shown in Fig. S1b, as expected. Meanwhile, when the plasmonic particles are immersed in the analyte (Fig. 5a of the main text) an improvement in the S_V is observed compared to the thin film case, as seen in Fig. S1c. This can be explained by the combination of the reduced volume with the increased interaction of the analyte with the field distributions of the excited LSP resonances, which translates into an improved sensitivity (Fig. 5e of the main text). However, when observing the S_V of the apertures filled with the analyte, shown in Fig. S1d, a large increase can be seen. This is due to the sensitivity of the filled apertures (unnormalized as shown in Fig. 5j from the main text) having values near the same order of magnitude of sensitivity as the thin film analyte (unnormalized as shown in Fig. 4f from the main text) while the volume of the analyte is two orders of magnitude less. For both cases of the plasmonic particles

and apertures being immersed and filled by the analyte, respectively, the plane wave polarization that excites a field hotspot between the plasmonic dimers have larger values of S_V as expected, E_y and E_x, respectively.

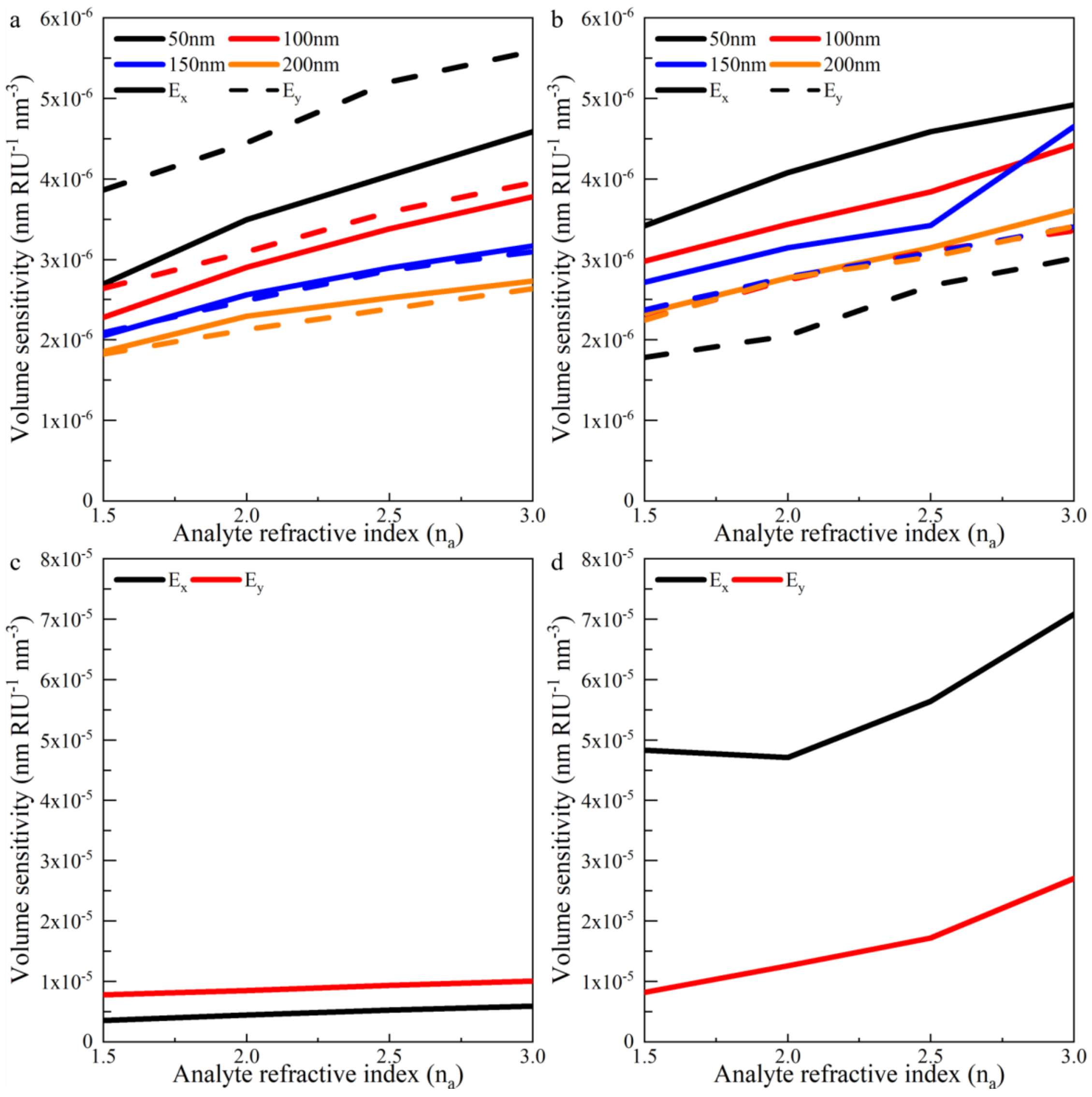

Figure S1. Volume sensitivity of the (a) plasmonic particles and (b) apertures for analyte thicknesses of δ_a = 50 nm (black), δ_a = 100 nm (red), δ_a = 150 nm (blue) and δ_a = 200 nm (orange). The dashed and solid lines correspond to the results using E_y or E_x illumination of the incident signal. Volume sensitivity of the (c) plasmonic particles and (d) apertures when the analyte surrounds/fills the plasmonic particles/apertures. The structures are illuminated by a plane wave polarised in the E_x (black) and E_y (red) direction. The y-scale of (a,b) and (c,d) are each different to better show the volume sensing performance of each configuration.

3. Sensitivity of plasmonic particles and apertures for small dielectric analytes

Here we consider the sensitivity and relative sensitivity per unit volume of the plasmonic particles and apertures when the volume of the dielectric analyte is significantly reduced. A schematic representation of the plasmonic particles with the analyte atop can be seen in Fig. S2a,b, on the *yz*- and *xy*-planes, respectively, with the schematic representations of the complementary plasmonic aperture shown in Fig. S2c,d. The dielectric analyte of dimensions 200 nm × 100 nm with a thickness of 50 nm (volume 1×10^6 nm^3) is positioned atop the plasmonic structures. The sensitivity of the particles (black) and apertures (red) under E_x (solid) and E_y (dashed) illumination is then calculated using the same method as Fig. 4,5 from the main text and the results are shown in Fig. S2e. As observed, the plasmonic structures that generate a field hotspot (i.e. plasmonic particles under E_y and plasmonic apertures under E_x illumination, produced significantly higher sensitivities, compared to modes without hotspots due to little interaction of excited mode with the analyte. In Fig. S2f the sensitivity per unit volume of the analyte is shown which is calculated using the same method as in Fig. S1. By comparing the results from Fig. S2e with those shown in Fig. 4 and Fig. 5 of the main text, the obtained sensitivities are significantly reduced, as expected due to the reduced volume of analyte. However, when comparing the sensitivity per unit volume shown in Fig. S2f and the values shown in Fig. S1, the values are comparable when a hotspot is excited (E_y and E_x illumination of the plasmonic particles and apertures, respectively).

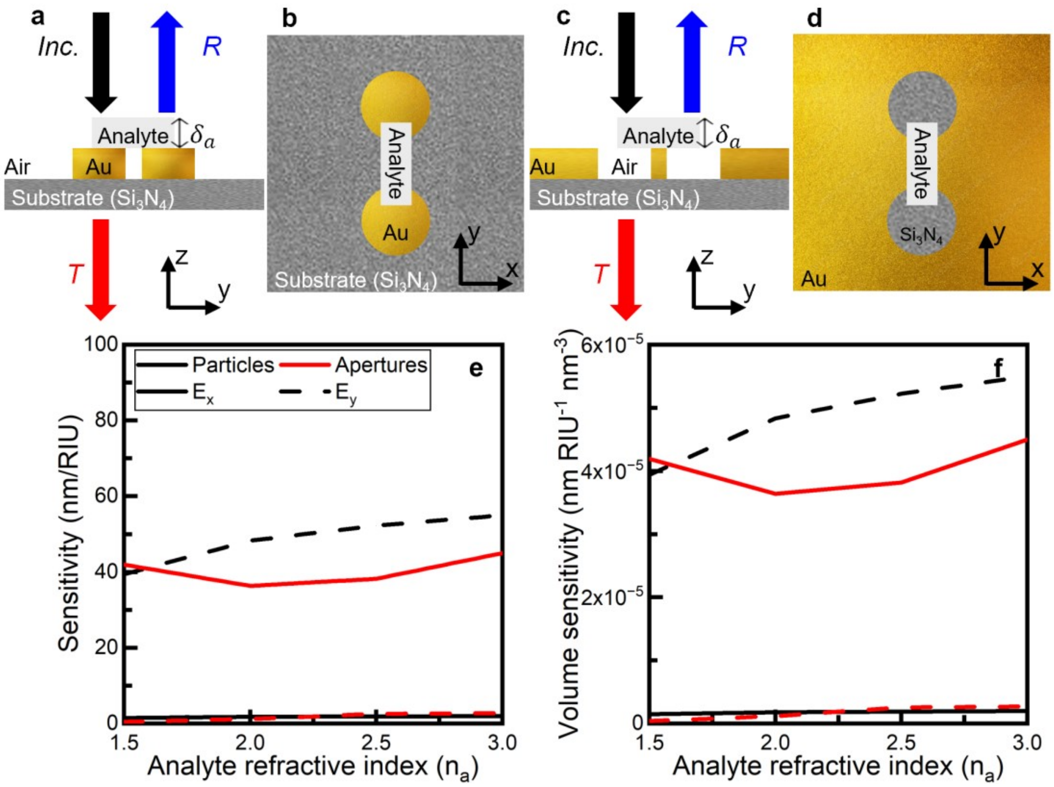

Fig. S2|. Sensitivity and sensitivity per unit volume of the plasmonic structures with an analyte block positioned atop. 2D Schematic representation of the cross-section on the (a,c) *yz*- and (b,d) *xy*-plane for plasmonic particles and apertures, respectively, used to determine changes of a nearby block of analyte with thickness of δ_a = 50 nm. (e) Sensitivity and (f) volume sensitivity of plasmonic particles (black) shown in (a,b), and plasmonic apertures (red) from (c,d) when n_a is changed from 1.5 to 3 in steps of 0.5 considering an incident plane wave with E_x (solid line) and E_y (dashed line) polarisation.
